# Terbinafine and antidepressants: Potential risk of medication induced mania

**DOI:** 10.1192/j.eurpsy.2021.528

**Published:** 2021-08-13

**Authors:** P. Barbosa, O. Nombora, J. Monteiro, L. Ribeiro

**Affiliations:** Psychiatric Department, Vila Nova de Gaia/Espinho Hospital Center, Vila Nova de Gaia, Portugal

**Keywords:** Terbinafine, Antidepressants, Pharmacological Interactions, Induced Mania

## Abstract

**Introduction:**

Mood destabilization and induced manic episodes are well-known phenomenon under antidepressant medications. However, even with a cautious introduction of antidepressants, it’s important to be aware of possible pharmacological interactions. Terbinafine is a known inhibitor of CYP2D6, a major hepatic metabolizer of a full list of antidepressant medications, and so capable of raising their serum levels and potentiating their side effects.

**Objectives:**

With this case report we aim to emphasize the importance of cautious usage of Terbinafine when combined with antidepressant medications.

**Methods:**

We present a clinical case of an induced first manic episode after the introduction of Terbinafine in a patient under antidepressant medication and a qualitative review on the topic, using PubMed database.

**Results:**

A 66-year-old woman, with an history of Major Depressive Disorder, previously medicated with Venlafaxine 75mg/day and Mirtazapine 30mg/day, was brought to the emergency department because of psychomotor agitation. She also had an history of seasonal fluctuating mood, although never fulfilling the criteria for Bipolar Disorder. At admission, her clinical status was compatible with a manic episode. This episode followed two months after the initiation of Terbinafine for onychomycosis.

**Conclusions:**

There are few studies that have shown antidepressant toxicity mediated by an interaction with Terbinafine. As far as we know this is the first case of induced mania after the introduction of Terbinafine. Therefore, it is important to remind that Terbinafine is a potential interacting agent when combined with psychotropic medications.

